# Relationships among fertility concerns, fear of cancer recurrence, social support, self-efficacy, and family resilience among Chinese adolescents and young adults with cancer: A structural equation modeling

**DOI:** 10.1371/journal.pone.0341351

**Published:** 2026-02-02

**Authors:** YuQiao Xiao, Can Gu, Li Liu, XiaoRou Zeng

**Affiliations:** Xiangya School of Nursing, Central South University, Changsha, People’s Republic of China; Tsinghua University, CHINA

## Abstract

Fertility concerns, fear of cancer recurrence, social support, and self-efficacy are key factors that influence family resilience in adolescents and young adults with cancer. However, their combined effects and underlying mechanisms remain unclear. This study constructed a structural equation model to examine these relationships and provide evidence for the development of targeted psychosocial interventions in clinical practice. A cross-sectional survey was conducted from April 2024 to March 2025 using convenience sampling of 259 adolescents and young adults with cancer at a tertiary-level specialized cancer hospital in Hunan Province. All the participants completed validated measures of fertility concerns, fear of cancer recurrence, social support, self-efficacy, and family resilience. A structural equation model was constructed using the AMOS software to test the hypothesized pathways. Correlation analysis revealed significant associations between all variables. The structural equation model demonstrated good fit. Path analysis revealed that fertility concerns and fear of recurrence exerted significant negative effects on family resilience, while social support and self-efficacy exerted significant positive effects. Further analysis revealed that social support and self-efficacy partially mediated the relationships between fertility concerns, fear of cancer recurrence, and family resilience. Family resilience among adolescent and young adult patients with cancer was significantly associated with fertility concerns, fear of cancer recurrence, social support, and self-efficacy. Social support and self-efficacy can mitigate and mediate the negative effects of fertility concerns and the fear of cancer recurrence on family resilience. Therefore, future clinical interventions should prioritize enhancing social support and self-efficacy among adolescents and young adults with cancer to alleviate fertility concerns and fear of cancer recurrence, thereby strengthening family resilience.

## Introduction

According to Global Burden of Disease data, cancer is the fourth leading cause of death among adolescents and young adults (AYAs), with approximately 40,380 new cases diagnosed annually in China, ranking it second worldwide in disease burden [1]. Advances in early screening and cancer treatment have enabled more AYAs to achieve long-term survival [2,3]. However, this population is at a unique developmental stage and faces challenges regarding education, career development, intimate relationships, and family planning. Compared to children or older adults, AYAs carry a heavier psychosocial burden. Family serves as the primary source of material and emotional support, and family functioning directly shapes a patient’s ability to adapt to illness and long-term psychosocial outcomes. Family resilience (FR), defined as the ability of family members to adapt, reorganize, and grow together in the face of adversity, plays a vital role in supporting AYAs [4]. Higher FR has been shown to help cancer patients and their families maintain emotional stability, improve treatment adherence, and enhance the quality of life [4,5].

Among the many factors influencing FR, fertility concerns (FC) are particularly salient in AYAs [6]. Standard treatments such as surgery, radiotherapy, and chemotherapy often cause reproductive and gonadal toxicity, imposing heavy burdens on patients’ identity, intimate relationships, and future family roles [7–10]. Fear of cancer recurrence (FCR) is defined as persistent worry about disease progression or relapse and is a common negative emotion [11]. Previous research suggests a strong co-occurrence between FC and FCR; fertility-related decisions may trigger recurrent fears, while high levels of FCR can intensify fertility distress [12]. This intertwined psychological dilemma not only impairs individual adaptation but also undermines family functioning [13–15].

Protective resources are crucial to buffer these adverse effects. According to the Family Adjustment and Adaptation Response (FAAR) model, external resources, such as social support (SS), and internal resources, such as self-efficacy (SE), significantly reduce illness-related stress. Higher perceived SS enhances coping capacity, while SE improves self-care and emotional regulation; together, they promote family functioning and adaptation [14–16]. Although previous studies have identified SS and SE as important predictors of FR, their mediating roles in the relationships among FC, FCR, and FR remain insufficiently explored.

**Fig 1:**
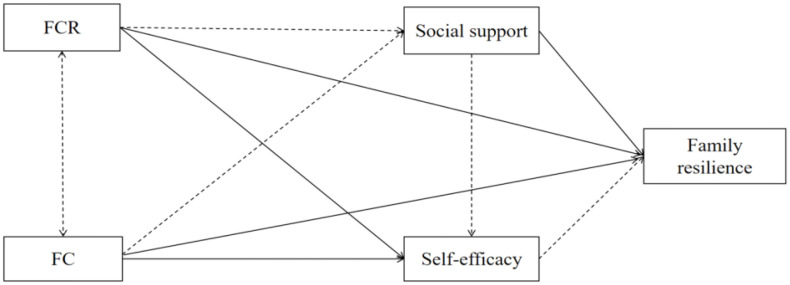
Research concept hypothesis diagram.

Despite the growing attention being paid to FR, existing research has largely focused on single psychological variables, with limited exploration of how multiple factors interact to influence resilience. In Chinese culture, where reproduction and family roles are highly valued, AYAs may face amplified challenges; however, empirical evidence is still lacking. Furthermore, few studies have applied structural equation modeling (SEM) to test these complex pathways. Therefore, this study aimed to construct and validate a SEM based on the FAAR model to examine the mechanisms linking FC, FCR, SS, and SE to FR. We propose the following hypotheses: (a) FC and FCR are positively correlated; (b) both factors negatively influence FR; and (c) SS and SE mediate the relationships between FC, FCR, and FR (Fig 1).

## Method

### Patients and settings

Adolescent and young adult cancer patients who met the inclusion criteria were recruited from April 2024 to March 2025 at a tertiary care hospital specializing in oncology in Hunan Province, China. The inclusion criteria were as follows: (a) female cancer patients aged 15–39 years, (b) conscious and able to cooperate with the investigation, and (c) those who voluntarily participated in this study and provided informed consent. The exclusion criteria were as follows: (a) patients with other comorbid diseases or cancers, (b) those with primary infertility, and (c) those with impaired verbal communication or slurred speech.

### Ethical considerations

All procedures were conducted in accordance with the Declaration of Helsinki of the World Medical Association and approved by the ethics committee of the investigator’s institution (Reference: E202430). Written informed consent was obtained from all participants prior to data collection. For participants under the age of 18, written consent was obtained from a parent or legal guardian in accordance with the ethical requirements.

### Measures

#### General information questionnaire.

A self-designed general information questionnaire was used to collect demographic and clinical data. These included demographic details, disease-related information, and fertility information.

#### Fear of Progression Questionnaire-Short Form (FOP-Q-SF).

Mehnert et al. [17] developed the FOP-Q-SF to measure fear of disease recurrence or progression in patients with cancer and chronic diseases. Mahendran et al. [18] adapted a Chinese version to evaluate the level of cancer-related fear among cancer survivors. The scale consists of 12 items, using a 5-point Likert scale, with total scores ranging from 12 to 60. The Cronbach’s α coefficient of the scale was 0.87.

#### Reproductive Concerns After Cancer scale (RCAC).

The RCAC was developed by Gorman et al. [19] to assess issues related to fertility and parenting among young adult female cancer survivors. The Chinese version of the scale was adapted from Qiao et al. [20]. The scale consists of six dimensions, with three items in each dimension. The Cronbach’s α coefficient for the scale was 0.792.

#### Multidimensional Scale of Perceived Social Support (MSPSS).

The MSPSS was developed by Zimet et al. [21] in 1988 and consists of three dimensions and 12 entries for friends, family, and significant others on a 7-point Likert scale with a total score range of 12–84. Scores of 12–36 indicates low, 37–60 indicates a moderate level of support, and 61–84 indicates a high level of support, scale was 0.890.

#### General Self-Efficacy Scale (GSES).

The General Self-Efficacy Scale (GSES) was originally developed by Schwarzer and Jerusalem [22] to assess individuals’ perceived ability to cope with a variety of challenging situations. The scale consists of 10 items and adopts a 4-point Likert response format, with higher scores indicating greater self-efficacy. The Chinese version of the GSES has demonstrated good internal consistency and validity across different samples, with reported Cronbach’s α coefficients ranging from 0.75 to 0.91 [23].

#### Family Hardiness Index (FHI).

The FHI was originally compiled by McCubbin et al. [24]. The Chinese version of the scale was translated, culturally adapted, and psychometrically validated by Liu et al. [25]. The scale has 20 items categorized into three dimensions: responsibility, control, and challenge. The total score ranges from 20 to 80, with higher scores indicating higher levels of family resilience. The scale demonstrated good reliability, with an overall Cronbach’s α of 0.803.

### Data collection

The study was conducted across five gynecological oncology wards, three breast wards, and one hematology ward, with the approval of the relevant institutions. The data collection process involved gathering sociodemographic information and patient responses to the three scales and extracting clinical data from medical records to ensure the integrity of the study. A research team was established before the formal survey began and standardized training was provided to all team members to ensure consistency. The questionnaire was administered in two formats: a paper questionnaire and an online questionnaire called Questionnaire Star. Patients first completed the paper questionnaire, which was then double-checked by two researchers before entering the Questionnaire Star system to identify invalid responses. A total of 280 questionnaires were distributed, and 21 invalid questionnaires were excluded, resulting in 259 valid responses. The actual recovery rate was 92.5%.

### Data analysis

We used the SPSS software (version 28.0) to analyze the data and calculate the frequencies, means, percentages, and standard deviations to characterize the sample. Pearson’s correlation coefficient was used to determine the relationships between the variables. Before conducting SEM, preliminary data screening was performed to assess the key statistical assumptions. Univariate normality was evaluated using skewness and kurtosis statistics, multivariate outliers were examined using Mahalanobis distance, and multicollinearity was assessed using variance inflation factor values. Minimal missing data were handled using full-information maximum likelihood. To assess potential common method bias, Harman’s single-factor test was conducted by entering all measurement items into an unrotated exploratory factor analysis. Confirmatory factor analysis was conducted to evaluate the measurement properties of the latent constructs, including FC, FCR, SS, SE, and FHI, by assessing factor loadings, internal consistency reliability, convergent validity, and discriminant validity. Subsequently, SPSS AMOS 24.0, was used to construct the structural model with FC, FCR, social support, self-efficacy, and FHI as latent variables and their corresponding items as observed indicators. Model fit was evaluated using multiple goodness-of-fit indices, including the goodness-of-fit index (GFI), the chi-square to degrees of freedom ratio (χ²/df), the root mean square error of approximation (RMSEA), the comparative fit index (CFI), and the incremental fit index. Direct, indirect, and total effects were estimated using SEM, and the bias-corrected bootstrap method with 5,000 re-samples was applied to test the significance of the mediating effects. A mediating effect was considered significant if the 95% bias-corrected confidence interval did not include zero [26]. In all analyses, a *p*-value < 0.05 was considered statistically significant.

## Results

### Participant characteristics

A total of 259 AYA cancer patients were investigated in this study, of which 147(56.8%) were under 28, 112 (43.2%) were over 29, 20 had a family history and 239 (92.2%) did not; 167 (64.5%) were married, 78 (30.1%) were unmarried, and 14 (5.4%) were divorced. The other general statistics are presented in Table 1.

**Table 1:** The participants’ general demographic and characteristics (*N* = 259).

Variables	Categories	n	%
General information			
Age (years)	16-28	147	56.8
	29-38	112	43.2
Nation	Han ethnic group	231	89.2
	Ethnic group	28	10.8
Religion	Yes	6	2.3
	No	253	97.7
Family history	Yes	20	7.7
	No	239	92.3
Residence	Rural	154	89.2
	Urban	105	10.8
Marital status	Married	167	64.5
	Unmarried	78	30.1
	Divorced	14	5.4
Educational level	Junior high school and below	55	21.2
	High School or College	63	24.3
	Bachelor’s degree or above	141	54.4
Disease information			
Cancer Type	Blood Cancer	17	6.6
	Breast Cancer	103	53.7
	Gynecologic Cancer	139	39.8
Cancer Stage	I	104	40.2
	II	79	30.5
	III	76	29.3
Treatment methods	Radiotherapy and chemotherapy	158	61.0%
	Endocrine	58	22.4%
	Surgery	43	16.6%
Fertility information			
Number of children	None	96	37.1
	1	88	34.0
	2 or more	75	29.0
Fertility intentions	Yes	141	54.4
	No	118	45.6

### Assessment of common method bias

The results of Harman’s single-factor test indicated that the first unrotated factor accounted for less than 50% of the total variance, suggesting that common method bias was not a serious concern.

### Relationships between fertility concerns, fear of cancer recurrence, social support, self-efficacy, and family resilience

Correlation analyses revealed that fertility concerns (r = –0.474, *p* < .001) and fear of cancer recurrence (r = –0.431, *p* < .001) were both significantly negatively correlated with family resilience. Social support (r = .486, p < .001) and self-efficacy (r = .468, *p* < .001) were positively correlated with family resilience. Further details are presented in Table 2. details.

**Table 2:** Correlations among fertility concerns, fear of cancer recurrence, social support, self-efficacy, and family resilience(N = 259).

Variables	Fertility concerns	Fear of Cancer Recurrence	Social Support	Self-Efficacy	Family Resilience
Fertility Concerns	1				
Fear of Cancer Recurrence	.285**	1			
Social Support	−.357**	−.345**	1		
Self-Efficacy	−.321**	−.306**	.393**	1	
Family Resilience	−.474**	−.431**	.486**	.468**	1

**P < 0.01.

### Structural equation modeling of AYAs cancer patients

**Fig 2:**
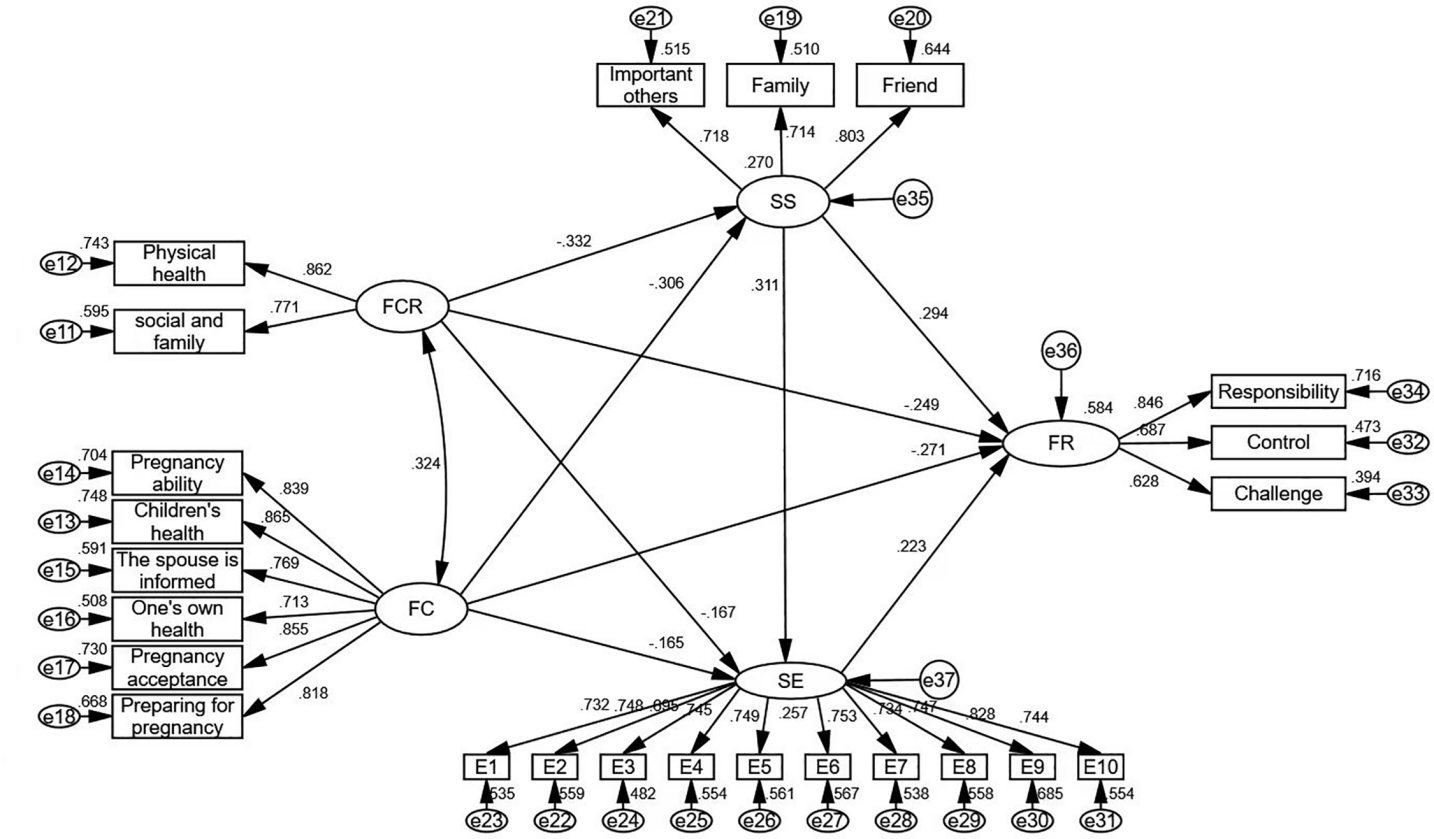
Structural equation modeling of AYAs cancer patients.

We constructed a structural equation model for AYAs with cancer. We hypothesized that fertility concerns and fear of cancer recurrence are exogenous variables, and social support, self-efficacy, and family resilience are endogenous variables. The results showed that all goodness of fits met the applicable index criteria (x2/df = 1.335; GFI = 0.908; RMSEA = 0.036; NFI = 0.912; TLI = 0.973; CFI = 0.976). This indicates that the structural equation model developed in this study fit well with the study data, as shown in Table 3 and Fig 2.

**Table 3:** Goodness-of-fit indices for structural equation modeling of AYAs cancer patients (N = 259).

Index	Absolute fit index				Relative fit index		
	x2	x2/df	GFI	RMSEA	NFI	TLI	CFI
Index standard		<3	>0.9	<0.08	>0.9	>0.9	>0.9
Model Result	323.047	1.335	0.908	0.036	0.912	0.973	0.976

Path analysis showed that FC had a negative effect on social support (β = −0.306, *P* < 0.001) and SE (β = −0.165, *P* < 0.05). FCR had a negative effect on SS (β = −0.332, *P* < 0.001) and self-efficacy (β = −0.167, *P* < 0.05). SS and SE positively influenced each other (β = 0.311, *P* < 0.001). SS (β = 0.294, *P* < 0.001) and SE (β = 0.233, *P* < 0.01) were positive influences on FR. Further details are presented in Table 4. details.

**Table 4:** Path analysis table for structural equation modeling of AYAs cancer patients.

Path	Unstandarderd coefficient	standarderd coefficient	*S.E.*	*C.R.*	*P*
FCR → SS	−0.484	−0.332	0.117	−4.147	***
FC → SS	−0.301	−0.306	0.073	−4.149	***
FCR → SE	−0.181	−0.167	0.083	−2.197	0.028
FC → SE	−0.121	−0.165	0.051	−2.376	0.018
SS → SE	0.232	0.311	0.063	3.708	***
SE → FR	0.144	0.223	0.044	3.267	0.001
SS → FR	0.142	0.294	0.039	3.599	***
FCR → FR	−0.175	−0.249	0.051	−3.428	***
FC → FR	−0.128	−0.271	0.032	−4.063	***

***P < 0.001.

### Chain-mediated effects of social support and self-efficacy

**Fig 3:**
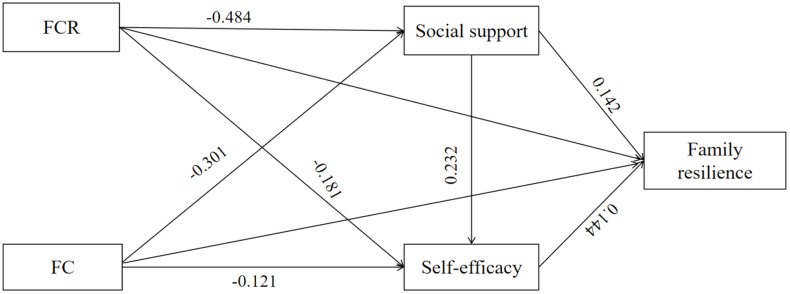
Research hypothesis verification diagram.

Mediation results showed that SS and SE played significant chain-mediated roles in FC, FCR, and FR. In particular, the effect value for mediating Effect 1 (FC-SS-SE-FR) was −0.023 (SE = 0.012, *p* = 0.004), and the effect value for mediating Effect 2 (FCR-SS-SE-FR) was −0.021 (SE = 0.011, *p* = 0.005). Further details are provided in Table 5 and Fig 3.

**Table 5:** Chain-mediated effects of social support and self-efficacy.

Path	Effect	*SE*	Bias-corrected 95%CI		*P*
			Lower	Upper	
FCR → SS → FR	−0.098	0.047	−0.218	−0.029	0.003
FC → SS → FR	−0.09	0.043	−0.193	−0.028	0.003
FCR → SE → FR	−0.037	0.025	−0.108	−0.004	0.028
FC → SE → FR	−0.037	0.024	−0.1	−0.004	0.018
FCR → SS → SE → FR	−0.023	0.012	−0.062	−0.007	0.004
FC → SS → SE → FR	−0.021	0.011	−0.055	−0.006	0.005

## Discussion

This study assessed the correlations among FC, FCR, SS, SE, and FR using structural equation modeling to validate the potential mediation among these factors. The results indicated that FC and FCR were significantly and negatively associated with FR, with SS and SE serving as mediators.

This study found a significant positive correlation between FC and FCR, which is consistent with previous findings. This result may be due to the fact that, when facing a major health crisis such as a cancer diagnosis, AYAs cancer patients reassess their self-worth and the value of life after going through a cancer diagnosis and treatment. They must continually overcome the tremendous shock of cancer diagnosis and adapt to the changes in their bodies [27,28]. The inevitable damage to fertility caused by cancer diagnosis and treatment methods may lead to a sense of resource plundering, particularly among young women, resulting in a pessimistic perception of fertility loss [12]. Influenced by traditional concepts, women are often expected to assume more reproductive roles within their family [10]. Additionally, AYA are at a critical stage of personal development. Due to the economic downturn and the unique social “inward spiral” in China, they must navigate the transformation of their social roles while constantly balancing career development, self-improvement, and family expectations. Consequently, when patients face both the threat of death from cancer and fertility issues, they often struggle to manage the relationship between survival and fertility, viewing them as contradictory and finding it difficult to maintain a balance in their decision-making processes [3,7].

The main findings of this study confirmed the chain-mediated roles of SS and SE. Specifically, excessive FC and FCR diminished patients’ perceived SS and SE. This reduction, in turn, negatively impacts their FR level and adversely affects their physical and mental health, as well as the quality of life of both patients and their family members. This is consistent with Lazarus’s theory of stress and coping, which states that when faced with a major crisis, individuals mobilize both internal and external resources to assess and cope with the stressor [29]. When stress is too high, it can weaken their resources and coping strategies, which in turn affect the outcomes of their physical, mental, and social dimensions of adaptation [29–31]. The results of the SEM pathway confirmed that SS and SE, as external and internal resources for individuals, can significantly alleviate the negative psychological burdens of FC and FCR. Previous studies have shown that a strong social support system can effectively alleviate negative psychological experiences in patients with cancer [32,33]. This enhances their ability to cope with crises, thereby increasing their confidence and sense of control in managing such events. This finding is consistent with the results of the present study. This suggests that in actual clinical intervention practice, the importance of social support and psychosocial outcomes for patients should be emphasized.

In addition, we found that peer support was important for AYAs with cancer. Peer support was the social support dimension with the highest scores. This may be because, under the influence of our traditional culture, young patients believe that reporting good news is a sign of filial piety and love for their parents. They tend to rely heavily on their families after an immediate illness but are reluctant to fully expose their vulnerabilities and sadness to their families in the early stages of the disease, which is not conducive to the overall resilience of the patient and their family. Therefore, friends provide vital spiritual support for patients with cancer. Pain associated with cancer diagnosis, treatment, and the disease itself often requires patients to invest more time and energy to cope with stress. This increased burden can lead to a decrease in social interactions and changes in friendships, which negatively impacts the physical and mental health of patients with cancer [34]. This suggests that we should pay attention to the significant role of peer support for AYA cancer patients, as well as the emotional changes and negative psychological burdens they experience. We can implement interventions such as lectures or live broadcasting classes, inviting patients of the same age during the recovery period to share inspirational stories about their fight against the disease. This approach provides strong informational support and emotional value, enhances self-efficacy in coping with illness, and strengthens family resilience.

### Limitations

Although this study confirmed the mediating role of social support and self-efficacy in the family resilience of cancer patients facing a major crisis by constructing a structural equation model, there are still some limitations. First, although this study defined a group of AYAs with cancer, the actual percentage of young patients with cancer was larger because data collection was conducted in only one hospital. Second, all data collected in this study were related to female patients with cancer, which might limit the general applicability of this research. Male cancer patients can also be included to explore the differences in the influence of related variables under different sexes, with the aim of enriching the research results further.

Furthermore, although statistical tests indicated no significant common method bias, the study relied entirely on self-reported measures, which may have introduced potential common method and reporting biases. Finally, this was a cross-sectional study, which could not interpret the dynamics of these variables, and the mediated outcomes need to be treated with caution. Therefore, in the future, we recommend a multicenter longitudinal study to better confirm the causal relationship between these variables.

## Conclusion

Perceiving strong social support and a powerful sense of self-efficacy are effective ways to help cancer patients cope with the fear of cancer recurrence and fertility concerns and enhance the resilience of their families. This suggests that, in future clinical work, the levels of social support and self-efficacy of patients should be identified early, effective intervention approaches should be established, and buffer pathways should be provided to patients to help them cope better with major crisis events.

## Supporting information

S1 Data Dataset. (XLSX)
